# Exploring factors underlying the attitude of community pharmacists to generic substitution: a nationwide study from Poland

**DOI:** 10.1007/s11096-015-0227-8

**Published:** 2015-11-30

**Authors:** Aleksandra Drozdowska, Tomasz Hermanowski

**Affiliations:** 1 Department of Pharmacoeconomics, Medical University of Warsaw, ul. Żwirki i Wigury 81, 02-091 Warsaw, Poland; 2 Department of Pharmacoeconomics, InterQuality Project Leader (7FP), The President of the Polish Society of Health Economic, Medical University of Warsaw, ul. Żwirki i Wigury 81, 02-091 Warsaw, Poland

**Keywords:** Educational campaigns, Generic substitution, Pharmacists’ knowledge, Pharmacists’ opinions, Poland, Quality, Safety, Therapeutic efficacy

## Abstract

*Background* Generic uptake will increasingly be promoted by governments in the face of increasing healthcare costs and global economic uncertainties. *Objective* The purpose of this study was to investigate attitudes towards generic substitution among community pharmacists, with a focus on the perception of the efficacy, knowledge of the generics characteristics, as well as the willingness to recommend generic substitution. *Setting* Community pharmacies in Poland. *Method* The survey was conducted in 2013 by telephone interviews with 802 holders of an MSc degree in pharmacy working as community pharmacists. Stratified sampling was implemented to make the study representative in geographic terms. *Main outcome measure* Pharmacists’ attitudes towards generics drugs. *Results* The study showed that only 40 % of pharmacists always inform patients about their right to choose a generic substitute. It was also shown that the less time a pharmacist has been practising, the less likely they are to invite consumers to choose between generic and innovator products. The likelihood of informing was not affected by pharmacist’s sex or age, or by pharmacy location or status (chain vs. independent pharmacy) (*p* > 0.05). Pharmacists varied in their approach to their statutory obligation to inform about a generic; a more or less equal share of respondents were either in favour or against it. Approximately 60 % pharmacists were shown to be familiar with the definition of a generic medicine. Pharmacists with shorter time of practice proved to know more about generics. However, more than 30 % respondents failed to choose the correct statement on generic versus reference medicine dosage. The majority of respondents (67 %) believed there are no differences in efficacy between generics and innovator drugs, whereas 31 % claimed that original brands could be more effective. A significant correlation was demonstrated between the views of pharmacists on the therapeutic efficacy and their willingness to substitute for generics whenever permitted by a physician. *Conclusion* It is important to address all concerns pharmacists may have over generics, for example by implementing comprehensive awareness-raising campaigns. Also, pharmacotherapy monitoring systems (i.e. provided in a framework of pharmaceutical care) could be considered to identify any safety or quality concerns that may arise.

**Electronic supplementary material:**

The online version of this article (doi:10.1007/s11096-015-0227-8) contains supplementary material, which is available to authorized users.

## Impacts on practice

- Pharmacists who are against the legal obligation to inform consumers about optional substitution also have a more skeptical opinion on the therapeutic efficacy of generics.
- An education campaign for pharmacists should be planned to refresh the knowledge on generic medicines of especially the older pharmacists.

## Introduction

During the 1980s and 1990s, most new legal regulations were intended to curtail the increasing costs of general medical care, with medicine being the major cost in healthcare globally [1]. Generally speaking, increasing drug expenditure over the past 10 years can be attributed to supply (the introduction of new expensive technologies) and demand (an ageing society and an increased prevalence of some health conditions). Due to the underlying assumption that market competition would not guarantee affordable prices alone, a wide range of regulatory interventions were implemented [2].

Generic drugs represent a major share in the drug market in Poland [1]. Drug expenditure in Poland is one of the lowest in Europe—$306 per capita in 2011 [3]. However, patient co-payment for drugs in Poland is the highest among all of the OECD countries, 60.8 % in 2009 with an increase of 3.4 % between 2000 and 2009 [4] (approximately 35 % of Poles reported that they could not afford to buy prescribed drugs and almost 8 % admitted to having resigned from or discontinued treatment for financial reasons [5]). According to the Polish Drug Reimbursement Act of May 2011, public spending on drug reimbursement cannot exceed 17 % of overall public spending on healthcare services guaranteed under the financial plan of the National Health Fund (NFZ).

Since EU accession, Polish legislative and regulatory framework has been harmonized with EU directives governing production, market placement, advertising and the marketing of medicinal products, in addition to relevant supervision and quality control regulations [1]. In Poland, GMP requirements for the pharmaceutical industry are governed by the Regulation of the Minister of Health of 1 October 2008 concerning Good Manufacturing Practice requirements (Journal of Laws Dz.U. 2008.184.1143).

The introduction of generic medicines to the market has a positive and statistically significant effect on reducing the prices of innovator drugs [7]. Furthermore, generics are believed to be a key factor in fuelling competition in the pharmaceutical market [8]. The price of medicines is not always determined by the balance between supply and demand. Currently, the pharmaceutical sector is heavily influenced by laws and regulations introduced by the government, such as official lists of reimbursed drugs, or detailed marketing authorization procedures. Pharmacies are obliged to inform customers that they can opt for a generic drug instead of the innovator medicine they have been prescribed; this should be indicated in written form in a visible and accessible location in the premises. Since the Drug Reimbursement Act of 1st January 2012, the duty to inform patients of generic substitution has been limited to reimbursement drugs only [9]. Polish drug pricing policy aims to add generics to the list of drugs eligible for reimbursement. Prescribing doctors still have the right to specify whether generic drug substitution is allowed and there is no obligation to include the international non-proprietary name (INN) in drug prescriptions [10].

The current definition of ‘generic medicinal products’ is found in Directive 2001/83/EC, Article 10(2)(b), which states that a generic medicinal product is a product which has the same qualitative and quantitative composition of active substances and the same pharmaceutical form as the reference medicinal product, and whose bioequivalence with the reference medicinal product has been demonstrated by appropriate bioavailability studies [6]. Tests are carried out according to the European guidelines and recommendations for bioavailability and bioequivalence studies (CPMP/EWP/QWP 1401/98), under which 90 % of confidence limits for Cmax and AUC should be between 80 and 125 %. In certain defined circumstances the 80–125 % range can be adjusted, for example, to address the concerns derived from literature reports describing the intensification of disease symptoms after switching NTI drug for a generic [11], the EMA introduced a tighter acceptance range for NTI drugs. EU guidelines define a 90.00–111.11 % acceptance range for the AUC of all NTI drugs. The Committee for Human Medicinal Products (CHMP) decides whether a particular drug meets the NTI drug criteria. For special efficacy and safety reasons, the CHMP can decide to reduce the C_max_ acceptance range to 90–111.11 %.

The main goal in bioequivalence testing is to detect variations in absorption and not test how the drugs work as they both contain the same active substance. Variations in absorption can be observed with each subsequent dose due to intra-subject variability. By definition, generic and branded drugs both contain exactly the same active substance at precisely the same dose. Thus, generic versus brand drug absorption would have to be tested for: possible absorption variations attributed to the presence of qualitatively and quantitatively different excipients or variations in technological procedures used in drug production and taking into account intra-subject variability.

Doing this can be challenging, as intra-subject variability can mask possible absorption variations of identical active molecules from two different tablets (generic vs. brand-name drugs). The testing method must be highly sensitive to detect variations (instead of conformities) in the absorption of the active substance from the intestines into the bloodstream; it must answer the question of whether absorption is unaffected by excipients. It is therefore essential that all mechanisms which transport the active substance through the intestinal wall are present in the test, including epithelial cell enzymes, transport proteins responsible for active substance delivery from the blood into the intestinal lumen and vice versa, as well as enzyme and transporter inhibitors in cells. This is why healthy, young individuals with normal intestinal wall functions are chosen to test bioequivalence [12].

Pharmacists play a key role in managing drug expenditure without losing therapeutic efficacy [13]. However, the role of a community pharmacist in selecting generics is complex [14–17]. Not only do they select a bioequivalent medicine, but they also educate consumers on issues around generic substitution, such as patient compliance. They also help to avoid patient confusion due to changes in brand medication and provide information on the quality and safety of generics to healthcare providers [14–17]. The attitude of consumers and pharmacists may be one barrier regarding the uptake of generics. Generics are perceived as less effective and less safe than innovator drugs. Therefore they are insufficiently used, which is the main reason why health care systems worldwide are ineffective, according to the World Health Organization [18].

### Aim of the study

This study aims to deliver baseline data to support the implementation of a generic substitution policy based on the perceptions and behaviour of pharmacists, and to evaluate views on generic medicines among community pharmacists in Poland. In order to do this, 802 pharmacists were investigated; their attitudes towards, knowledge of and willingness to substitute generic medicines for innovator drugs were investigated. Pharmacists’ perceptions of the efficacy of generic medicines and how pharmacists feel about the current national policy on generic substitution was also analysed.

### Ethical approval

The manuscript does not contain clinical studies or patient data. The Ethics Committee of the Medical University of Warsaw does not require consent (and does not issue opinions) for this kind of research [19].

## Method

### Sampling and study representativeness

A description of the study including a detailed sampling method to make the study as representative as possible as well as observational error estimations are presented below.

Sampling consisted of selecting individual units (pharmacies) to make up the statistical sample, representative for the general population. Stratified sampling was selected to enable the best geographic representativeness of the study. In stratified sampling, the general population is broken down into strata and then independent samples are randomly selected from each separate stratum. Stratification was based on the location of pharmacies and the whole of Poland was divided into 16 different regions. A list of pharmacies in Poland, with a special focus on their location (i.e. their province), was used as the sampling frame.

The size of individual strata was determined from the actual numerical distribution of pharmacies throughout Poland. According to the Central Statistical Office, there were 11,999 community pharmacies in Poland in 2012. Table 1 presents data on the number of community pharmacies in each region. The number of interviews to be conducted with pharmacists in individual regions was calculated from a sample size of 802 units, whose structure was identical to that of the general population. Moreover, the study was performed according to pre-defined sampling assumptions to make it representative in all regions.

**Table 1:** Sampling

Sampling strata—regions	General population		Study sample	
	Number of community pharmacies	Structure in %	Number of community pharmacies	Structure in %
Lodzkie province	864	7.20	58	7.23
Masovia province	1585	13.21	107	13.34
Lesser Poland province	1112	9.27	75	9.35
Silesia province	1395	11.63	93	11.60
Lublin province	805	6.71	54	6.73
Subcarpathia province	600	5.00	40	4.99
Podlaskie province	352	2.93	23	2.87
Holy Cross province	417	3.48	28	3.49
Lubusz province	304	2.53	20	2.49
Greater Poland province	1137	9.48	76	9.48
West Pomerania province	502	4.18	33	4.11
Lower Silesia province	970	8.08	65	8.10
Opole province	303	2.53	20	2.49
Kuyavia-Pomerania province	583	4.86	39	4.86
Pomerania province	682	5.68	45	5.61
Warmia-Masuria province	388	3.23	26	3.24
Poland	11,999	100	802	100

*Source*: based on the data from Local Data Bank of the Central Statistical Office in Poland (www.stat.gov.pl) and on the study results

### Observational error

The acceptable observational error in a representative study was determined according to relevant statistical rules and relates to population size, study sample size, and the acceptable confidence level. The study covered all community pharmacies operating in Poland. Given the subject matter of the study—generic substitution—respondents were pharmacists who held an MSc in pharmacy and worked at one of the selected pharmacies.

The acceptable observational error for the sample size used in this study was calculated using the following formula:

$$ n = \frac{{P\left( {1 - P} \right)}}{{\frac{{e^{2} }}{{Z^{2} }} + \frac{{P\left( {1 - P} \right)}}{N}}} $$

where: P—estimated proportion in the general population—a standard value of 50 %; e—maximum acceptable observational error (calculated); n—study size (802); N—population size (11,999); Z—Z value was calculated from the confidence level (1.96 at 95 % confidence level).

The study was conducted for 95 % confidence level and 50 % fraction per population value. Maximum observational error was estimated at 3.34 %. Observational error is the maximum acceptable difference between the estimated value of a parameter determined from a sample and the true value in the population concerned. Therefore, actual values may differ from the study results by up to 3.34 percentage points.

### Interviews

The survey was conducted in October 2013 by telephone interviews with 802 holders of MSc degrees in pharmacy working at community pharmacies. These were structured (standardized) interviews, i.e. the interviewer asked a pre-defined list of questions.

The questionnaire used during the interviews was tested for face and content validity by two public opinion research experts, and adjusted after pilot tests with 50 pharmacists. The final questionnaire included demographic questions as well as specific questions concerning the experience and opinions of pharmacists in the area of generic substitution. The attitudes of pharmacists to various aspects of generic substitution were analysed on a five-point Likert scale. Respondents remained anonymous.

### Statistical methods

Statistical analysis was performed using SPSS Statistics v.21 (IBM). Because the Kołmogorow–Smirnow test demonstrated statistically significant deviations from the normal distribution of quantitative variables, nonparametric equivalents were used. Correlations between variables were measured in the Spearman’s rho rank correlation analysis, and the differences between mean values were calculated with the Mann–Whitney *U* test. Nominal variables were measured using the Chi square test.

## Results

The Mann–Whitney *U* test (Table 2) demonstrated a significant difference between less experienced pharmacists (practising for 1–5 years) compared to more experienced pharmacists (11–15 years) (U = 9628.5, *p* = 0.013); less experienced pharmacists were less likely to inform consumers about the availability of cheaper generic substitutes. A significant difference was also confirmed for pharmacists with 1–5 versus 16–20 years of practice (U = 8610.5, *p* = 0.001) and those with 1–5 versus ≥21 years of practice (U = 9925.5, *p* = 0.001).

**Table 2:** Frequency of sharing information on generic substitution among the study population (n = 802)

Sociodemographic characteristics	Total (n)	Q1: How often do you inform consumers that they can buy a cheaper generic instead of the prescribed innovator product (assuming that both products are available for sale at the pharmacy)? n (%)				
		Never	Rare	Sometimes	Often	Always
*Gender*						
Female	700 (100 %)	0 (0 %)	24 (3.4 %)	66 (9.4 %)	326 (46.6 %)	284 (40.6 %)
Male	102 (100 %)	0 (0 %)	6 (5.9 %)	13 (12.7 %)	41 (40.2 %)	42 (41.2 %)
*Age*						
25–34 y	245 (100 %)	0 (0 %)	7 (2.9 %)	35 (14.3 %)	113 (46.1 %)	90 (36.7 %)
35–44 y	312 (100 %)	0 (0 %)	11 (3.5 %)	21 (6.7 %)	150 (48.1 %)	130 (41.7 %)
45–54 y	182 (100 %)	0 (0 %)	9 (4.9 %)	14 (7.7 %)	81 (44.5 %)	78 (42.9 %)
55–64 y	51 (100 %)	0 (0 %)	1 (2 %)	8 (15.7 %)	20 (39.2 %)	22 (43.1 %)
65 y and over	12 (100 %)	0 (0 %)	2 (16.7 %)	1 (8.3 %)	3 (25 %)	6 (50 %)
*Pharmacy status*						
Chain pharmacy	262 (100 %)	0 (0 %)	11 (4.2 %)	32 (12.2 %)	108 (41.2 %)	111 (42.4 %)
Independent pharmacy	540 (100 %)	0 (0 %)	19 (3.5 %)	47 (8.7 %)	259 (48 %)	215 (39.8 %)
*Years of practice as a pharmacist*						
1–5	184 (100 %)	0 (0 %)	12 (6.1 %)	33 (18 %)	78 (42.6 %)	61 (33.3 %)
6–10	235 (100 %)	0 (0 %)	12 (5.1 %)	18 (7.7 %)	117 (49.8 %)	88 (37.4 %)
11–15	129 (100 %)	0 (0 %)	3 (2.3 %)	12 (9.3 %)	54 (41.9 %)	60 (46.5 %)
16–20	118 (100 %)	0 (0 %)	1 (0.8 %)	5 (4.2 %)	61 (51.7 %)	51 (43.2 %)
20 and more	136 (100 %)	0 (0 %)	3 (2.2 %)	11 (8.1 %)	57 (41.9 %)	65 (47.8 %)
*Pharmacy location*						
Urban area of over 500,000 inhabitants	138 (100 %)	0 (0 %)	6 (4.3 %)	17 (12.3 %)	64 (46.4 %)	51 (37 %)
Urban area of 100,000–500,000 inhabitants	184 (100 %)	0 (0 %)	4 (2.2 %)	16 (8.7 %)	97 (52.7 %)	67 (36.4 %)
Urban area of up to 100,000 inhabitants	387 (100 %)	0 (0 %)	14 (3.6 %)	38 (9.8 %)	162 (41.9 %)	173 (44.7 %)
Rural area	93 (100 %)	0 (0 %)	6 (6.5 %)	8 (8.6 %)	44 (47.3 %)	35 (37.6 %)
Total	802 (100 %)	0 (0 %)	30 (3.7 %)	79 (9.9 %)	367 (45.8 %)	326 (40.6 %)

Other factors—including sex, age, pharmacy status (chain vs. independent pharmacy), and pharmacy location—did not differentiate the respondents to a statistically significant extent in terms of how frequently they informed customers about their generic option (*p* > 0.05).

Sixty-seven percent of respondents believed the efficacy of cheaper generics was no worse than that of innovator medicines, and around 3 % of respondents believed it may be superior. Nearly 30 % of pharmacists claimed generics were sometimes less effective; only 1 % claimed that generics were typically less effective (Table 3, Q2).

**Table 3:** Attitude of pharmacists towards generics (n = 802)

Survey question/answers	n	%
Q2: Do you think generics are:		
Typically less effective than innovator medicines	6	0.9
Sometimes less effective than innovator medicines	233	29
Equally effective as innovator medicines	538	67
Sometimes more effective than innovator medicines	22	2.7
Typically more effective than innovator medicines	3	0.4
Q3: When buying drugs yourself, you typically choose:		
Generics	201	25
Either a generic or a innovator medicine	530	66
Innovator medicines	71	9

If they were to buy a medicine for themselves, 25 % of respondents stated that they would choose a cheaper generic drug, whereas 9 % expressed a preference for innovator products. The majority of pharmacists (66 %) had no a priori preferences and would decide on a case by case basis (Table 3, Q3).

A significant correlation between the perception of pharmacists for the therapeutic efficacy of generic versus innovator medicines (Q2) and their positive attitude to generic substitution (Q4) was demonstrated in the Spearman’s rho rank correlation analysis (Rho = 0.21, *p* < 0.001). The correlation was positive, i.e. pharmacists who believed in the efficacy of generics were more likely to be in favour of generic substitution, whenever the code ‘NZ’ (indicating ‘do not substitute’) is absent from a prescription.

Two-thirds of pharmacists were in favour of generic substitution unless contraindications existed (i.e. a “do not substitute” note on the prescription). 32 % of respondents were in favour and 32 % opposed imposing a legal obligation on pharmacists to inform consumers about the availability of generics (Table 4).

**Table 4:** Responses of pharmacists to questions exploring their perceptions of generic policy (n = 802)

Survey question/statement	n (%)				
	Strongly disagree	Disagree	Neutral	Agree	Strongly agree
Q4: I am in favour of generic substitution (whenever the code ‘NZ’ is absent from the prescription)	13 (1.6 %)	45 (5.6 %)	206 (25.7 %)	396 (49.4 %)	142 (17.7 %)
Q5: I believe pharmacists should be legally bound to inform consumers about the generic substitute of the prescribed innovator medicine	93 (11.6 %)	160 (20.0 %)	293 (36.5 %)	173 (21.6 %)	83 (10.3 %)

A closer look was taken at those respondents who were against imposing a legal obligation on pharmacists to inform consumers of their generic option. These pharmacists were shown to be more sceptical with regard to the therapeutic efficacy of generics compared to innovator drugs (Q2 vs. Q5, U = 24,669, *p* < 0.001).

Likewise, those against generic substitution considered generics to be less effective than innovator products compared to those in favour of generic substitution unless contraindications existed (Q2 vs. Q4, U = 10,976, *p* < 0.001).

**Fig. 1:**
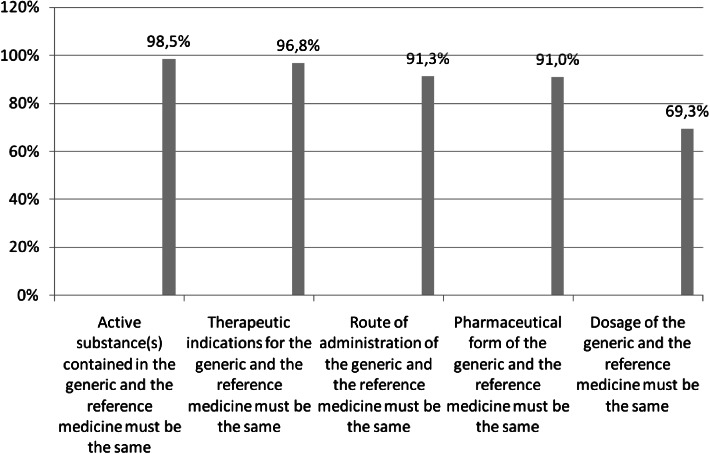
Percentage of pharmacists who agreed on the following statements based on the EMA definition of a generic medicine (Q6, all statements were correct) (n = 802)

Out of 802 respondents, 507 had a full understanding of the European Medicines Agency (EMA) definition of generics, i.e. they marked all five statements correctly that define a generic product in Question 6 [20, 21]. Almost all respondents agreed that the active substance(s) and therapeutic indications of a generic and the originator brand must be identical (99 and 97 % respectively). The vast majority of pharmacists knew that the pharmaceutical form and route of administration for generics and the originator brands had to be the same (91 %). However, 31 % of respondents were not aware that the dosage of generics and originator brands must also be the same (Fig. 1).

During the next stage of analysis, respondents were divided into two groups: the first group demonstrated only a partial knowledge of the proper definition of generic medicines and included those pharmacists who selected 1–4 out of the 5 statements defining a generic in Question 6. The second group included those respondents who correctly recognised all 5 criteria of a generic medicine (Table 5).

**Table 5:** Time of practice vs. knowledge of the definition of generics (Q6, n = 802)

	Years of practice intervals					Total
	1–5 years	6–10 years	11–15 years	16–20 years	21 years and more	
Partial knowledge	43 (14.6 %)	90 (30.5 %)	51 (17.3)	64 (21.7 %)	47 (15.9 %)	295 (100 %)
Full knowledge	141 (27.8 %)	145 (28.6 %)	78 (15.4 %)	54 (10.7 %)	89 (17.5 %)	507 (100 %)
Total	184 (22.9 %)	235 (29.3 %)	129 (16.1 %)	118 (14.7 %)	136 (17.0 %)	802 (100 %)

A significant association was found to exist between respondents being able to fully define a generic medicine and their years of practice as a pharmacist (χ^2^(4) = 30.28, *p* < 0.001). Respondents with 1–5 years of practice were more familiar with all definition criteria. The longer respondents were in practice, the less likely they were to be familiar with all criteria defining a generic. Respondents with only partial knowledge of the definition of generics had typically been practicing for 6–20 years (Table 5).

Knowledge of all five criteria defining a generic medicine (Q6, full vs. partial knowledge) did not differentiate the respondents to a statistically significant extent in terms of how frequently pharmacists informed consumers about the availability of generic medicines (Q1, *p* > 0.05) or in terms of being for or against generic substitution (Q4, *p* > 0.05).

## Discussion

This study shows that the majority of pharmacists in Poland always (40 %) or often (46 %) inform consumers about a generic option. Pharmacists with more professional experience are more likely to inform customers, this was also confirmed in another recent Polish study carried out in Lodzkie province [22]. Factors such as: sex, age, pharmacy status (chain pharmacy vs. independent pharmacy), and pharmacy location did not differentiate the respondents to a statistically significant level in terms of frequency with which they made consumers aware that they could opt for generic substitution (*p* > 0.05). Chong et al. [23] arrived at similar conclusions after testing 500 randomly selected Australian pharmacies from across the country. He reported no significant differences in the frequency of recommending generic substitution between urban and rural areas, or between pharmacists who worked at different types of pharmacies (e.g. independently owned, banner group). In this study, pharmacists stated that, when possible, they offered generic substitutes for almost all (96.4 %) innovator products prescribed [23].

In 2012, generics accounted for a 70 % market share in Germany and around 60–68 % in Poland, the United Kingdom, the Netherlands and Denmark (other European countries had a lower generic market share) [24]. Poland is a country with a mature generic medicine market, however, the percentage share of pharmacists who always offered generic substitution was higher in some other countries [25, 26]. This could be explained by the poor enforcement of the statutory obligation to inform patients about generic substitution. Additionally, this study has revealed that pharmacists in Poland have different opinions on whether they should be legally bound to inform patients that a cheaper equivalent is available.

Nearly one-third of all respondents were against imposing a legal obligation on pharmacists to inform consumers about generics, which may explain why some pharmacists have been reluctant to adhere to these laws. Pharmacists who were against this legal obligation were shown to have a more negative attitude to the therapeutic efficacy of generic versus brand name drugs. Therefore, negative attitudes (and the resulting potential non-compliance) to the legal obligation to inform consumers of generic availability may be attributed, at least to some extent, to the poor perception of generic efficacy. The uptake of generics could be greater if these pharmacists had more positive attitudes to the therapeutic efficacy of generic drugs.

In Chong et al.’s 2010 study in Australia [26], 93.7 % of respondents declared they were ready to offer generic substitutes unless it was explicitly forbidden. In Poland, this figure was less than 70 %. Australia has implemented several education campaigns among pharmacists, which may have contributed to the high percentage of pharmacists in favour of generic substitution. One example being the National Prescribing Service, the Pharmaceutical Society of Australia, and the Pharmacy Guild of Australia work together and now supply information and guidelines on generic medicines to community pharmacists [27]. These education initiatives are aimed to consolidate knowledge and confidence among pharmacists to teach consumers about how to safely and appropriately use generics. For instance, a “Generic medicines are an equal choice” campaign was launched in 2008, where a generics tool kit was sent to each community pharmacist [28]. The tool kit contained practical guides on brand substitution and ancillary labels with which pharmacists found it easier to inform consumers about the active ingredients of dispensed medicines [29]. Due to the positive effects of these actions, similar measures could be contemplated in Poland or elsewhere. Moreover, since August 2008, pharmacists in Australia are encouraged to dispense cheaper brands by being paid a financial incentive whenever they offer a substitutable, premium-free PBS medicine (the incentive is AUD 1.50 as of August 2010) [30].

Around 63 % of pharmacists in Poland had full knowledge of the definition of generics. Analysis revealed something particularly noteworthy—pharmacists in practice for longer were less able to fully define generic medicine. The same result was found in a 2012 study, on a group of 625 pharmacists from New Zealand, by Babar et al. [31]. However, both in Babar et al., and in this study, better knowledge does not necessarily prompt pharmacists to support generic substitution, and may not alter actual dispensing habits.

In this study, the majority of respondents (67 %) reported no difference in efficacy between generics and the originator brands, whereas 31 % claimed that original brands could be more effective than generics. Similarly high percentages of pharmacists who believe in the higher efficacy of innovator medicines can be observed in other countries. In Babar et al.’s study, around 50 % of respondents believed originator brands were more effective, and around 70 % of respondents recognised generics as bioequivalent to reference innovator medicines. In another study, by Chong et al. [13], among Malaysian community pharmacists, 21 % of all respondents supported the statement that generic medicines were of inferior quality and only around half of them believed that generics are therapeutically equivalent to the innovator drug. The results of the study by Babar et al. [31] and Chong et al. [13], suggest that the negative opinion of at least part of the respondents concerning the quality and efficacy of generics may perhaps be attributed to the fact that a large percentage of those pharmacists were in both cases unaware (or questioned the fact) that any product approved as a generic equivalent had to be bioequivalent to the originator drug by definition. In this study, almost one-third of respondents believed in the occasional superiority of innovator medicines. Future research should investigate the factors underlying the negative perceptions of some Polish pharmacists towards generics to discover whether this is due to personal prejudices or negative patient feedback.

## Conclusion

This study delivers baseline data to support improvements to the generic substitution policy in Poland, however, conclusions could also be relevant to decision-makers from other EU countries.

In order to be clinically based, the decision whether to substitute should be grounded in appropriate medical evidence. Therefore, clear-cut guidelines and recommendations for safe generic substitution, which prescribing physicians or pharmacists could rely upon, are essential. Specifically, a guide for health professionals describing therapeutically equivalent and non-equivalent medicinal products, such as the Orange Book in the US, in order to contain the risk of errors and irregularities around generic substitution should be created. Guidelines of this kind should be developed at a national level, based on the relevant domestic legislation [32].

The main focus should be on the education of health professionals. It is their awareness and sensitivity to warning symptoms that is decisive for the effectiveness of Adverse Drug Reaction (ADR) reporting. Comprehensive awareness-raising campaigns should therefore be considered. If the impact of such education initiatives is evaluated, it will also be easier to investigate the causes of negative perceptions of generic efficacy, such as personal prejudice or negative patient feedback.

Finally, pharmacotherapy monitoring systems in a framework of pharmaceutical care should be considered to ensure that any safety or quality concerns could be easily identified. In the future, pharmacists may find it easier to monitor the safety of pharmacotherapy by relying on an integrated IT system, which provides access to patient’s medical history and treatment, used by outpatient clinics, hospitals, and pharmacies. This will help identify possible therapy-related risks.

## Electronic supplementary material

Below is the link to the electronic supplementary material.

Supplementary material 1 (PDF 422 kb)
