# Supplementary material for: Exploring factors underlying the attitude of community pharmacists to generic substitution: a nationwide study from Poland
Source: Int J Clin Pharm. 2015 Nov 30;38(1):162–70. doi: 10.1007/s11096-015-0227-8 (PMC4733132; doi:10.1007/s11096-015-0227-8)
Supplement: Supplementary file 1 — Supplementary material 1 (PDF 422 kb) [file 11096_2015_227_MOESM1_ESM.pdf]

Questionnaire

Part I Sociodemografic data

**1. M.Sc. in pharmacy/pharmacology**<br>a. yes<br>b. no	**2. Pharmacy status**<br>a. chain pharmacy<br>b. independent pharmacy
**3. Age**<br>a. 25-34 y<br>b. 35-44 y<br>c. 45-54 y<br>d. 55-64 y<br>e. 65 y and over	**4. Gender**<br>a. female<br>b. male
**5. Pharmacy location**<br>a. urban area of over 500 000 inhabitants<br>b. urban area of 100.000 - 500.000 inhabitants<br>c. urban area of up to 100,000 inhabitants<br>d. rural area	**6. Years of practice as a pharmacist**<br>a. 1-5<br>b. 6-10<br>c. 11-15<br>d. 16-20<br>e. 20 and more

Part II Specific questions

Q1: How often do you actively inform patients of generic substitutes for the prescribed innovator equivalents?

a. never
b. rarely
c. sometimes
d. often
e. always

Q2: Do you think generics are:

a. Typically less effective than innovator medicines.
b. Sometimes less effective than innovator medicines.
c. Equally effective as innovator medicines.
d. Sometimes more effective than innovator medicines.
e. Typically more effective than innovator medicines.

Q3: When buying drugs yourself, you typically choose:

a. generics
b. either a generic or a innovator medicine
c. innovator medicines

Q4: I am in favour of generic substitution, whenever the code 'NZ' is absent from the prescription:

a. strongly disagree
b. disagree
c. neutral agree
d. strongly agree

Q5: I believe pharmacists should be legally bound to inform consumers about the generic substitute of the prescribed innovator medicine

a. strongly disagree
b. disagree
c. neutral agree
d. strongly agree

Q6:. Please select all true statements. It is a multiple choice question:

a. Active substance(s) contained in the generic and the reference medicine must be the same
b. Therapeutic indications for the Generic and the reference medicine must be the same
c. Route of administration of the Generic and the reference medicine must be the same
d. Pharmaceutical form of the Generic and the reference medicine must be the same
e. Dosage of the generic and the reference medicine must be the same

Q7: Please indicate the importance of the following factors if you were considering to inform the patient of generic substitutes for the prescribed innovator equivalents.

**Statement**	1 – 'irrelevant'	2 – 'small relevance'	3 – 'moderate relevance'	4 – 'high relevance'	5 – 'very high relevance'
cost-saving potential for patients					
past experience with generic substitutes					
type of condition (chronic/acute)					
customer volume at the pharmacy and the resulting lack of time to explain generic-related issued to patients					
legal regulations					
concern about poor patient compliance associated with generic substitution					
concern about impact on relationship with patient when generic medicine does not work					
